# Single Nucleotide Polymorphisms in the Tumor Necrosis Factor-Alpha Gene Promoter Region Alter the Risk of Psoriasis Vulgaris and Psoriatic Arthritis: A Meta-Analysis

**DOI:** 10.1371/journal.pone.0064376

**Published:** 2013-05-23

**Authors:** Junqing Zhu, Hongda Qu, Xiaoguang Chen, Hao Wang, Juan Li

**Affiliations:** 1 Department of Rheumatology, Nanfang Hospital, Southern Medical University, Guangzhou, Guangdong, China; 2 Department of Traditional Chinese Internal Medicine, School of Traditional Chinese Medicine, Southern Medical University, Guangzhou, Guangdong, China; 3 Department of Traditional Chinese Medicine Diagnosis, School of Traditional Chinese Medicine, Southern Medical University, Guangzhou, Guangdong, China; 4 Key Laboratory of Prevention and Control for Emerging Infectious Diseases of Guangdong Higher Institutes, Department of Pathogen Biology, School of Public Health and Tropical Medicine, Southern Medical University, Guangzhou, Guangdong, China; University of Leicester, United Kingdom

## Abstract

**Background:**

It has been confirmed that tumor necrosis factor-alpha (TNFα), a macrophage-derived pro-inflammatory cytokine, plays an important role in the pathogenesis of psoriasis vulgaris and psoriatic arthritis (PsV&PsA). In contrast, the reported association of TNFα gene promoter region single nucleotide polymorphisms (SNPs) and PsV&PsA has remained controversial. Accordingly, we performed a meta-analysis to provide new evidence that SNPs in the TNFα gene promoter region alter not only the risk of psoriasis vulgaris (PsV) or psoriatic arthritis (PsA) but also of PsV&PsA.

**Methods:**

Interrelated literature dated to October 2012 was acquired from the PubMed, ScienceDirect, and SpringerLink databases. The number of the genotypes and/or alleles for the TNFα promoter in the PsV and PsA and control subjects was obtained. Odds ratios (ORs) and 95% confidence intervals (CIs) were used to calculate the risk of PsV and/or PsA with TNFα promoter SNPs.

**Results:**

A total of 26 papers of 2159 for PsV (2129 normal controls) and 2360 for PsA (2997 normal controls) were included in our meta-analysis. The results showed that the variant genotype and allele of TNFα -308A/G was protective in pooled groups of patients with PsV&PsA (OR = 0.682, 0.750; 95% CI, 0.596-0.779, 0.653-0.861). However, the variant genotypes and alleles of TNFα -238A/G and -857T/C had an increased risk of PsV&PsA (OR = 2.493, 2.228, 1.536, 1.486, 95% CI, 1.777-3.498, 1.628-3.049, 1.336-1.767, 1.309-1.685). Moreover, the meta-analysis revealed a significant association between TNFα -238A/G and -857T/C polymorphism and PsA susceptibility (OR = 2.242, 2.052, 1.419, 1.465; 95% CI, 1.710-2.941, 1.614-2.610, 1.214-1.658, 1.277-1.681). In contrast, the variant genotypes and alleles of TNFα -308A/G proved to be protective against PsV (OR = 0.574, 0.650, 95% CI, 0.478-0.690, 0.556-0.759), whereas TNFα -238A/G was found to have a risk association (OR = 2.636, 2.223, 95% CI, 1.523-4.561, 1.317-3.751).

**Conclusions:**

SNPs in the TNFα gene promoter region alter the risk of PsV and/or PsA.

## Introduction

Psoriasis is a common autoimmune disorder that primarily involves the skin (psoriasis vulgaris, PsV). Severe complications, including generalized involvement of the body (erythroderma), extensive pustular lesions, and an associated arthritis known as psoriatic arthritis (PsA), make psoriasis complex and heterogeneous [1]. The worldwide prevalence of PsV varies from 0.91% (United States) to 8.5% (Norway) in adults [2]. However, only 34.4% of patients with PsV also have arthritis, with the onset of skin lesions preceding arthritis in 64.5% of the cases and arthritis appearing after PsV in only 19.35% of cases. In the remaining 16.1% of cases, PsV and PsA began almost simultaneously [3]. Thus, the relationship between the onset of PsV and PsA included in the spondyloarthritis group is closely related, although the exact pathogenesis has yet to be identified. The diseases are multifactorial and result from the interplay between multiple genetic, immunologic, and environmental factors. Under the category of genetic and environmental effects, cytokines, chemokines, adhesion molecules, and growth factors combined with T cells and dendritic cells act in an integrated manner to evolve into unique inflammatory and proliferative processes in psoriasis vulgaris and psoriatic arthritis (PsV & PsA) [1].

Tumor necrosis factor-alpha (TNFα), a macrophage-derived pro-inflammatory cytokine, has been proven to play an important role in the pathogenesis and development of PsV & PsA. By inducing and recruiting multiple cytokines, TNFα activates the inflammatory process of PsV & PsA in the skin, joints, and bowel [4]. The marked clinical response of TNFα blockers in patients has demonstrated the importance of TNFα in both PsV and PsA [5]. The TNFα gene has been localized to the major histocompatibility complex (MHC) region on chromosome 6p21.3, which is 250 kb centromeric from the human leukocyte antigen-B and thought to be a high-priority candidate gene in PsV & PsA [6]. Single nucleotide polymorphisms (SNPs) are common in the TNFα gene, especially in promoter region, and act as markers of disease susceptibility because of their influence on TNFα expression by a cooperative manner [7]. Kaluza et al. found that the psoriasis-associated TNFα promoter allele TNF238.2 showed a significantly decreased transcriptional activity and less tumor necrosis factor alpha production [8]. Although many studies have investigated the relationship between promoter region SNPs in the TNFα gene and the susceptibility to rheumatoid arthritis (RA), ankylosing spondylitis (AS), Crohn’s disease, PsV, and PsA [9]–[13], the association between TNFα promoter SNPs and PsV & PsA risk have produced inconsistent results. Some studies have found a significant association between TNFα promoter SNPs and PsV & PsA, whereas others have failed to confirm these results [14], [15]. Two meta-analyses, one of nine studies, the other of ten, were performed in 2006 and 2007, respectively, to evaluate the association between TNFα promoter SNPs and the susceptibility to PsA and PsV [11], [13]. However, because of the complexity and heterogeneity of disease, the association between PsV and PsA on pathogenesis is still unclear [16]. Thus, it is necessary to pool the results of these studies and systematically analyze the relationship between TNFα promoter SNPs and PsV & PsA risk. Moreover, new studies about the association between TNFα promoter SNPs and the susceptibility to PsV & PsA have recently been published and provide new evidence that was not included in the previous meta-analyses, particularly regarding the susceptibility alleles TNFα -857T/C, TNFα -1031C/T, and TNFα -863A/C [14], [15], [17]–[21]. Therefore, it is essential to collect the most comprehensive evidence and reassess the association of TNFα promoter SNPs with PsV and/or PsA.

## Materials and Methods

### Search strategy

We searched the PubMed, ScienceDirect, and SpringerLink databases for papers published up to October 2012. An electronic search was performed using combinations of the following search terms without any limits: “tumor necrosis factor-alpha”, “TNFα”, “promoter”, “polymorphisms”, “single nucleotide polymorphism”, “genotype”, “psoriatic arthritis”, “PsA”, and “psoriasis”. Reference lists were checked to identify repeated literature.

### Inclusion and exclusion criteria

For selection in the analysis, each study met all of the following criteria: (1) clinical subtypes of psoriasis, including PsV and/or PsA, according to the Moll and Wright or CASPAR criteria or diagnosed by clinical specialists; (2) a case-control or cohort study; (3) the inclusion the distributions of genotypes and/or alleles within the TNFα promoter provided for both the patients and controls; and (4) the reporting of the basic characters of the participants, including ethnicity, age and gender. The exclusion criteria were as follows: (1) a case report, review or descriptive study; (2) clinical subtypes of psoriasis, including erythroderma or extensive pustular lesions; (3) a lack of normal population as controls; (4) duplicate data in the studies; and (5) an inability to obtain the necessary data.

### Quality assessment

The quality assessment of the selected studies was performed independently by two authors using the Newcastle-Ottawa Scale (NOS) [22]. Three parameters of quality, selection, comparability, and exposure assessment were assessed for the case-control studies. We defined “low-quality studies” as those with scores of 1–3, “moderate-quality studies” as those with scores of 4–6, and “high-quality studies” as those with scores of 7–9. Any disagreements were resolved by discussion.

### Data extraction

The following information was extracted from each study: first author, publication year, country of origin, patient ethnicity and demographic characteristics, subtypes of psoriasis (PsV type I, II, and/or PsA), the number of cases and controls, the number of genotypes and/or alleles for the TNFα promoter in the cases and controls, and consistency tests of genotype frequencies with the Hardy-Weinberg equilibrium model (HWE). Two authors analyzed each paper according to the inclusion and exclusion criteria and independently extracted the information. In some studies, only the novel data were extracted because a portion of the data had already been reported. Disagreements were resolved by discussion.

### Statistical analysis

The risks of the variant genotypes and alleles compared with the wild-type for six sites of TNFα promoter SNPs were estimated separately. Different subgroups divided by ethnicity or disease subtype were analyzed. Odds ratios (ORs) and 95% confidence intervals (CIs) were used to calculate the risks of PsV and/or PsA with TNFα promoter SNPs. A p value <0.05 was considered statistically significant. In the forest plots, OR>1 represented a risk effect and <1 a protective effect. In our meta-analysis, the heterogeneity among the studies was tested by a χ^2^-based Q test and I^2^ statistic [23]. When p<0.10 or I^2^>50%, the heterogeneity was considered significant. A fixed-effects model was a better choice to calculate the pooled ORs when the heterogeneity was not significant, otherwise a random-effects model was used because of the heterogeneity among the studies. To explore the source of heterogeneity, a meta-regression analysis was performed when the number of studies was more than ten in a group for which heterogeneity existed [24]. A p value <0.05 was considered statistically significant. The size of the heterogeneity source (SH) was assessed by percentage. Both Begg’s and Egger’s tests were performed to check the publication bias [25]; the publication bias was considered significant when p<0.10 in either test. All of the analyses were conducted using Stata software version 11.0 (Stata Corporation, College Station, TX, USA).

## Results

### Characteristic analysis of the included literature and quality assessment

**Figure 1:**
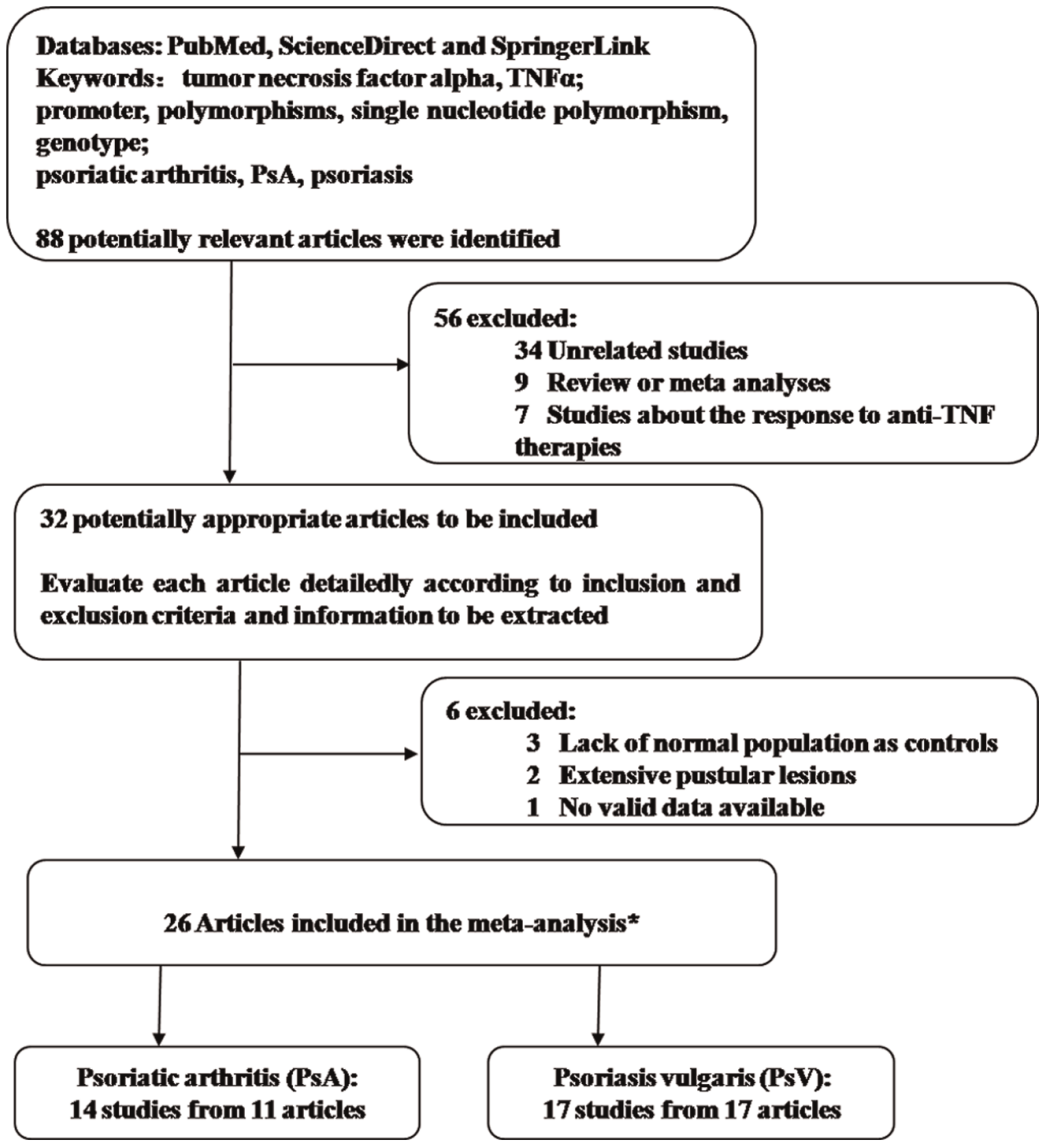
Flow diagram of the selection and nature of the studies. *including a meta-analysis of PsA with two studies available; both PsV and PsA were involved in the two articles.

According to the inclusion and exclusion criteria, 26 papers regarding the association between TNFα promoter SNPs and the risk of PsV & PsA, including 14 studies for PsA [13], [17], [20], [21], [26]–[32] and 17 studies for PsV[8], [14], [15], [18], [19], [21], [29], [32]–[42], were included in our analysis (Figure 1). Twenty-five studies (80.6%) were of high quality and six (19.4%) of moderate quality based on the NOS. In our meta-analysis, 2159 PsV (controls, 2129) reports and 2360 PsA reports (controls, 2997) were included to analyze the disease risk from TNFα -308A/G (rs1800629), -238A/G (rs361525), -857T/C (rs1799724), -1031C/T (rs1799964), -863A/C (rs1800630), and -488A/G (rs80267959) polymorphisms. Details about the first author, publication year, country of origin, ethnicity, subtype of psoriasis, number of cases and controls, number of genotypes and/or alleles for the TNFα promoter in the cases and controls, consistency tests of genotype frequencies with HWE, and the NOS score are shown in Tables 1 and 2 for each study selected. The publishing date of all the studies ranged from 1997 to 2012. All of the patients were Caucasian, with the exception of five studies that involved Asian patients. Consistency tests of genotype frequencies with HWE were performed in 8 (47.1%) and 9 (64.3%) studies of PsV and PsA, respectively.

**Table 1:** Characteristics of the individual studies for PsA included in the meta-analysis.

First author	Year	Country/Ethnicity	Type of psoriasis	SNPs:case/control (n)						HWE	NOS score
				308A/G	238A/G	857T/C	1031C/T	863A/G	488A/G		
Giardina-G	2011	Germany/Caucasian	PsA	-	-	370/560	-	-	-	Y	7
Giardina-I	2011	Italy/Caucasian	PsA	-	-	400/400	-	-	-	Y	7
Giardina-U	2011	UK/Caucasian	PsA	-	-	125/354	-	-	-	Y	7
Popa	2011	Romania/Caucasian	PsA	86/142	86/147	86/142	-	-	-	Y	7
Reich1	2007	Germany/Caucasian	PsA	361/370	368/372	370/373	367/368	-	-	Y	7
Rahman-N	2005	Newfoundland/Caucasian	PsA	225/103	228/103	220/103	220/103	231/103	-	Y	7
Rahman-T	2005	Toronto/Caucasian	PsA	203/101	199/101	199/101	200/99	200/101	-	Y	7
Balding	2003	Ireland/Caucasian	PsA	149/390	-	-	-	-	-	N	6
Gonzalez-S	2002	Spain/Caucasian	PsA	81/110	81/110	-	-	-	-	N	8
Al-Heresh	2002	Ireland/Caucasian	PsA	124/101	124/101	-	-	-	124/101	N	8
Hohler-1	2002	Germany/Caucasian	PsA	87/99	87/99	-	-	-	-	Y	7
Gonzalez-J	2001	Jewish/Caucasoid	PsA	52/73	52/73	-	-	-	-	N	8
Hamamoto	2000	Japan/Asian	PsA	20/87	20/87	20/87	20/87	20/87	-	N	8
Hohler-2	1997	Germany/Caucasian	PsA	62/99	62/99	-	-	-	-	Y	7

**Notes:** “PsA”, psoriatic arthritis; “SNPs”, single nucleotide polymorphisms; “HWE”, Hardy–Weinberg equilibrium; “NOS”, Newcastle-Ottawa Scale; “Y”, yes, consistency tests of HWE were performed; “N”, no, consistency tests of HWE were not performed. “-”, no statistics.

**Table 2:** Characteristics of the individual studies for PsV included in the meta-analysis.

First author	Year	Country/Ethnicity	Type of psoriasis	SNPs: case(type I/type II)/control (n)				HWE	NOS score
				308A/G	238A/G	857T/C	1031C/T		
Gallo	2012	Spain/Caucasian	PsV with or without PsA	84/76	81/71	77/76	86/72	N	6
Magalhaes	2010	Brazil/Caucasian	PsV type I	69(69/0)/70	69(69/0)/70	-	-	Y	8
Settin	2009	Egypt/Caucasian	PsV type I/type II	46/98	-	-	-	N	7
Nedoszytko	2007	Poland/Caucasian	PsV type I/type II	166/65	166(134/32)/65	-	-	Y	7
Reich1	2007	Germany/Caucasian	PsV	368/370	373/372	374/373	370/368	Y	7
Baran	2006	Poland/Caucasian	PsV type I/type II	78(54/24)/74	-	-	-	N	7
Mossner	2005	Germany/Caucasian	PsV with or without PsA	239/135	239/135	-	-	Y	6
Long	2004	China/Asian	PsV type I/type II	77/82	77(48/29)/82	-	-	N	7
Tsunemia	2003	Japan/Asian	PsV	163/96	163/96	-	-	N	7
Kim	2003	Korea/Asian	PsV type I/type II	103/125	103/125	-	-	N	6
Chang	2003	China/Asian	PsV type I/type II	105/160	105/160	-	-	N	7
Reich2	2002	Germany/Caucasian	PsV type I/type II	231/345	231(156/75)/345	-	-	Y	7
Hohler1	2002	Germany/Caucasian	PsV type I	60(60/0)/99	60(60/0)/99	-	-	Y	7
Craven	2001	UK/Caucasian	PsV type I/type II	81(48/33)/66	-	-	-	N	7
Kaluza	2000	Germany/Caucasian	PsV type I with or without PsA	47/43	47(47/0)/43	-	-	N	6
Reich3	1999	Germany/Caucasian	PsV type I/type II	151(100/51)/123	151(100/51)/123	-	-	Y	8
Jacob	1999	Germany/Caucasian	PsV type I	80(80/0)/99	83(83/0)/99	-	-	Y	6

**Notes:** “PsV”, psoriasis vulgaris; “SNPs”, single nucleotide polymorphisms; “HWE”, Hardy–Weinberg equilibrium; “NOS”, Newcastle-Ottawa Scale; “Y”, yes, consistency tests of HWE were performed; “N”, no, consistency tests of HWE were not performed. “-”, no statistics.

### Quantitative synthesis and heterogeneity analysis

1. TNFα -308A/G, -238A/G, -857T/C and -1031C/T polymorphism and susceptibility to PsV & PsA

**Figure 2:**
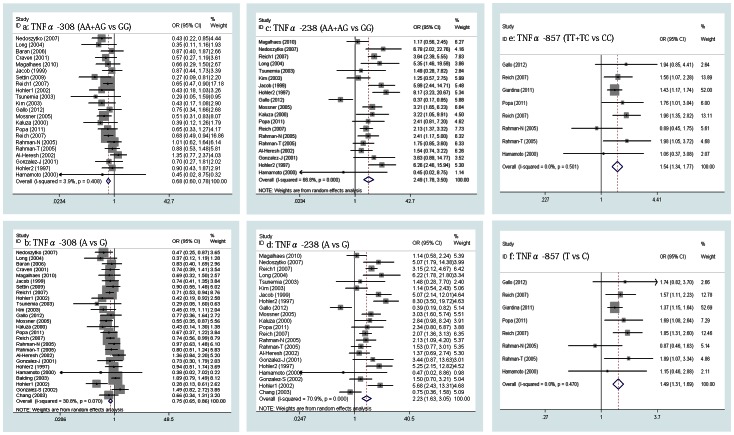
Forrest plot of the relationship between the TNFα promoter SNPs and risk of PsV & PsA. a–b, Pooled lower risk of PsV & PsA with TNFα -308A/G polymorphism; c-f, Pooled higher risk of PsV & PsA with TNFα -238A/G and TNFα -857T/C polymorphism.

We pooled all the studies involving both PsV and PsA to evaluate the disease risk associated with TNFα -308A/G, -238A/G, -857T/C and -1031C/T polymorphisms. The meta-analysis showed a significant association between the two genotypes of TNFα -308A/G (AA+AG vs GG) and PsV & PsA in a pooled group of 22 studies (OR = 0.682, 95% CI, 0.596-0.779, p = 0.000, Figure 2 a) with the fixed-effects model (Q = 21.85, p = 0.408, I^2^ = 3.9%), which suggested that the variant genotype of TNFα -308A/G (AA+AG) was protective. For the two alleles of TNFα -308A/G (A vs G), the same protective effect (OR = 0.750, 95% CI, 0.653-0.861, p = 0.000, Figure 2 b) was found with the random-effects model (Q = 36.13, p = 0.070, I^2^ = 30.8%) because of the heterogeneity among the 26 studies. To explore the heterogeneity source, a meta-regression analysis was performed, and the result indicated that the concomitant variable, the psoriasis subtype, had statistical significance (p = 0.041, SH 56.0%) while the publication year and ethnicity had no statistical significance (p>0.05). A subgroup analysis was performed.

However, the overall meta-analysis involved 19 and 22 studies, which separately indicated that the variant genotype (AA+AG) and allele (A) of TNFα -238A/G exhibited an increased risk of PsV & PsA (OR = 2.493, 2.228, 95% CI 1.777-3.498, 1.628-3.049, p = 0.000, 0.000 [separately], Figure 2 c, d) in the random-effects model (Q = 54.27, 72.07, p = 0.000, 0.000, I^2^ = 66.8%, 70.9% [separately]). The result of the meta-regression analysis showed that the publication year had statistical significance (p = 0.007, 0.014, SH 53.7%, 35.8% [separately]) while ethnicity and psoriasis subtype had no statistical significance (p>0.05).

Similarly, for the genotypes (TT+TC vs CC) and alleles (T vs C) of TNFα -857T/C, the pooled ORs were found to be 1.536 (95% CI, 1.336-1.767, p = 0.000, Figure 2 e) and 1.486 (95% CI, 1.309-1.685, p = 0.000; Figure 2 f) with the fixed-effects model for each group in eight studies (Q = 6.34, 6.62, p = 0.501, 0.470, I^2^ = 0.0%, 0.0% [separately]).

Our meta-analysis did not reveal a significant association between the TNFα -1031C/T polymorphism and susceptibility to PsV & PsA. The pooled ORs were 0.894 (95% CI, 0.755-1.059, p = 0.195) for the genotype with the fixed-effects model (Q = 8.27, p = 0.142, I^2^ = 39.6%) and 0.867 (95% CI, 0.693-1.084, p = 0.211) for the allele with the random-effects model (Q = 10.22, p = 0.069, I^2^ = 51.1%). A meta-regression analysis was not performed for <10 studies involved in the group in which heterogeneity existed.

2. TNFα -308A/G, -238A/G, -857T/C, -1031C/T, -863A/C, and -488A/G polymorphism and susceptibility to PsA

**Figure 3:**
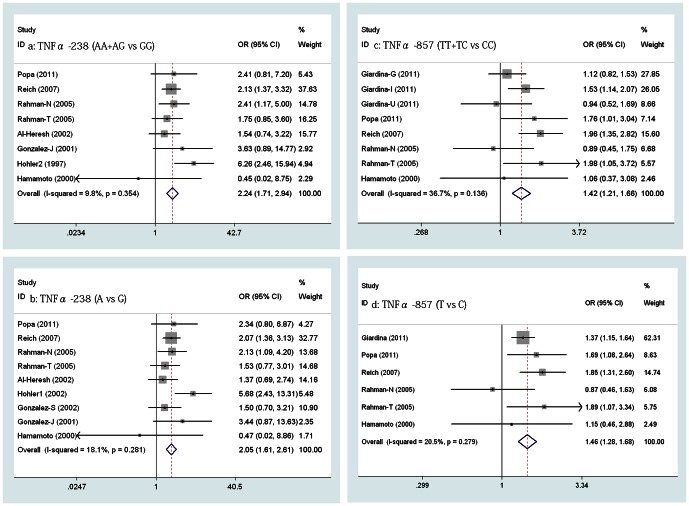
Forrest plot of the relationship between TNFα promoter SNPs and risk of PsA. a–d, Pooled higher risk of PsA with TNFα -238A/G and TNFα -857T/C polymorphism.

The meta-analysis showed a significant association between the two genotype of TNFα -238A/G (AA+AG vs GG) and PsA in eight pooled studies that included one study involving an Asian group from Japan [31] (OR = 2.242, 95% CI, 1.710-2.941, p = 0.000, Figure 3 a). Similarly, the two alleles of TNFα -238A/G (A vs G) were also correlated with the risk of PsA in the pooled analysis that added another study from Spain [27] (OR = 2.052, 95% CI, 1.614-2.610, p = 0.000; Figure 3 b). Even when the data from Japan was removed, the pooled OR also was statistically significant at 2.284 (95% CI, 1.731-3.004, p = 0.000) and 2.079 (95% CI, 1.632-2.649, p = 0.000 [separately]). All of the ORs stated above were calculated using the fixed-effects model, as there was no significant heterogeneity among the studies in every pooled analysis (Q = 7.76, 9.77, 6.63, 8.82, p = 0.345, 0.281, 0.357, 0.266, I^2^ = 9.8%, 18.1%, 9.5%, 20.7% [separately]).

The pooled OR from the eight studies including one Asian group [31] was first calculated to be 1.419 (95% CI, 1.214-1.658, p = 0.000) using the fixed-effects model (Q = 11.05, p = 0.136, I^2^ = 36.7%), which indicated that there was a significant association between the genotypes of TNFα -857T/C (TT+TC vs CC) and PsA (Figure 3 c). Additionally, a statistical significance was found for the two alleles of TNFα -857T/C (T vs C) (OR = 1.465, 95% CI, 1.277-1.681, p = 0.000, Figure 3 d) using the fixed-effects model (Q = 6.29, p = 0.279, I^2^ = 20.5%). Excluding the Asian group, the pooled ORs were not significantly affected (OR = 1.428, 1.473, 95% CI, 1.220-1.671, 1.281-1.693, p = 0.000, 0.000 [separately]).

Our meta-analysis did not reveal a significant association between TNFα -308A/G, -1031C/T and -863A/C polymorphism and the susceptibility to PsA. Together with the results of heterogeneity and publication bias tests, the values of ORs and 95% CIs for the risk of susceptibility in patients with PsA are shown in Table 3. All of the ORs were calculated using the fixed-effects model, although one pooled OR for the comparison of the TNFα -308A/G allele (A vs G) between PsA and the controls was observed using the random-effects model (Q = 19.20, p = 0.038, I^2^ = 47.9%). No pooled OR was performed for the TNFα -488A/G polymorphism, as only one study was involved.

**Table 3:** Summary of odds ratios (95% CI) in the analysis of the relationship between TNF-a promoter SNPs and PsA susceptibility.

Comparison	No. of studies	Odds Ratio (95% CI)	Test for OR		Test for heterogeneity			Publication bias	
			Z	P	Chi-squared	P	I^2^ (%)	Begg's Test	Egger's test
TNFα-308^ *(1)^	8	0.829(0.681, 1.008)^$^	1.88	0.06	5.88	0.554	0.00%	0.621	0.861
TNFα-308^* (2)^	7	0.831(0.683, 1.012)^ $^	1.84	0.065	5.72	0.456	0.00%	0.881	0.55
TNFα-308^# (1)^	11	0.876(0.704, 1.091)^&^	1.18	0.236	19.2	0.038	47.90%	0.392	0.489
TNFα-308^#(2)^	10	0.879(0.703,1.100)^&^	1.13	0.26	18.87	0.026	52.30%	0.531	0.586
TNFα-1031^* (1)^	4	0.854(0.685, 1.064)^ $^	1.4	0.16	2.35	0.504	0.00%	1	0.657
TNFα-1031^* (2)^	3	0.858(0.686, 1.074)^ $^	1.33	0.183	2.29	0.319	12.50%	0.602	0.264
TNFα-1031^# (1)^	4	0.860(0.715, 1.034)^ $^	1.6	0.109	2.29	0.514	0.00%	1	0.686
TNFα-1031^# (2)^	3	0.863(0.716, 1.040)^ $^	1.54	0.122	2.25	0.325	11.10%	0.602	0.316
TNFα-863^* (1)^	3	0.764(0.538, 1.084)^ $^	1.51	0.132	0.26	0.877	0.00%	0.602	0.733
TNFα-863^# (2)^	3	0.774(0.569,1.053)^ $^	1.63	0.103	0.47	0.789	0.00%	0.602	0.587

Notes:* The variant genotype; # the variant allele; (1) pooled group, including Asian and Caucasian groups; (2) pooled group including a Caucasian group only; & data from the random-effects model because of significant heterogeneity; $ data from the fixed-effects model.

3. TNFα -308A/G, -238A/G, -857T/C and -1031C/T polymorphism and susceptibility to PsV

**Figure 4:**
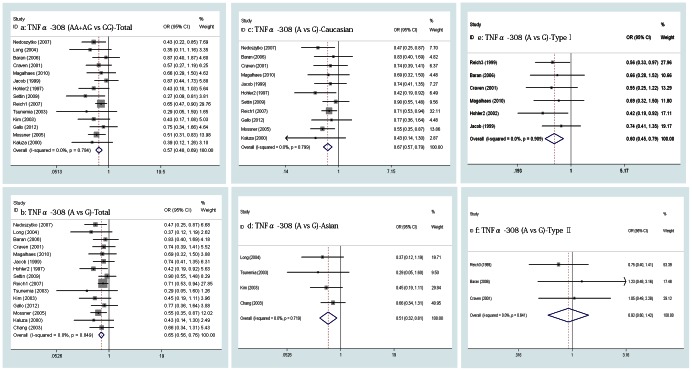
Forrest plot of the relationship between TNFα -308A/G polymorphism and risk of PsV, a–b, Pooled lower risk of PsV with TNFα -308A/G polymorphism; c–d, In Caucasian and Asian subgroups, pooled lower risk of PsV with TNFα -308A/G polymorphism; e, Pooled lower risk of type I PsV with TNFα -308A/G polymorphism; f, no risk of type ΠPsV with TNFα -308A/G polymorphism.

A meta-analysis was performed on all the PsV patients and subgroups divided by ethnicity and subtype of PsV, revealing a significant association between the two genotypes of TNFα -308A/G (AA+AG vs GG) and PsV in 14 pooled studies (OR = 0.574, 95% CI, 0.478-0.690, p = 0.000, Figure 4 a), which suggested that the variant genotypes of TNFα -308A/G (AA+AG) are a protective effect. The same association was revealed in both of the Caucasian and Asian subgroups in eleven and three studies, respectively, but the pooled OR was 0.596 for the Caucasian subgroup (95% CI, 0.492-0.721, p = 0.000) compared with 0.382 for the Asian subgroup (95% CI, 0.196-0.747, p = 0.005). For the two alleles of TNFα -308A/G (A vs G), the same protective effect was found in the pooled groups mentioned above. The OR values were 0.650 (95% CI, 0.556-0.759, p = 0.000), 0.672 (95% CI, 0.569-0.793, p = 0.000) and 0.507 (95% CI, 0.317-0.812, p = 0.005) (Figure 4 b, c, d). The pooled ORs in the type I subgroup (involving six studies) were 0.596 (95% CI, 0.437-0.813, p = 0.001) and 0.597 (95% CI, 0.450-0.791, p = 0.000; Figure 4 e) for the genotypes and the alleles of TNFα -308A/G (separately). However, the results for the genotypes and the alleles of TNFα -308A/G in the type II subgroups involving three studies were not statistically significant in indicating a protective effect (OR = 0.856, 0.923, 95% CI 0.523-1.400, 0.598-1.423, p = 0.535, 0.715 [separately], Figure 4 f). In all of the subgroups, the fixed-effects model was used to calculate the ORs because no heterogeneity was found among the studies (p>0.1, I^2^<50.0%).

**Figure 5:**
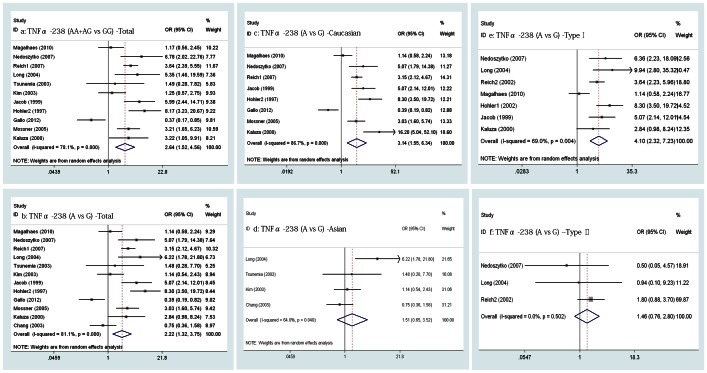
Forrest plot of the relationship between TNFα -238A/G polymorphism and risk of PsV. a–b, Pooled higher risk of PsV with TNFα -238A/G polymorphism; c–d, In Caucasian and Asian subgroups, pooled higher risk of PsV with TNFα -238A/G polymorphism; e, Pooled higher risk of type I PsV with TNFα -238A/G polymorphism; f, no risk of type ΠPsV with TNFα -238A/G polymorphism.

The overall meta-analysis involving 11 and 12 studies separately indicated that the variant genotypes (AA+AG) and allele (A) of TNFα -238A/G had an associated increase risk of PsV (OR = 2.636, 2.223, 95% CI, 1.523-4.561, 1.317-3.751, p = 0.001, 0.003 [separately], Figure 5 a, b) using the random-effects model (Q = 45.76, 58.23, p = 0.000, 0.000, I_2_ = 78.1%, 81.1% [separately]). To explore the source of heterogeneity, a meta-regression analysis was performed, and the result showed that the concomitant variable of publication year had a statistical significance (p = 0.02, 0.042, SH 54.8%, 37.1% [separately]) while ethnicity, type of PsV and HWE consistency test had no statistical significance (p>0.05). However, we also calculated the pooled ORs in the subgroups divided by ethnicity and type of PsV, and the same risk of the variant genotypes (AA+AG) and allele (A) of TNFα -238A/G was found in all the subgroups with the existence of heterogeneity using the random-effects model, which was not found using the fixed-effects model in subgroups of type II PsV (Figure 5 c, d, e, f).

No pooled OR was calculated for the TNFα -857T/C and -1031C/T polymorphisms, as only two studies were involved.

### Publication bias

**Figure 6:**
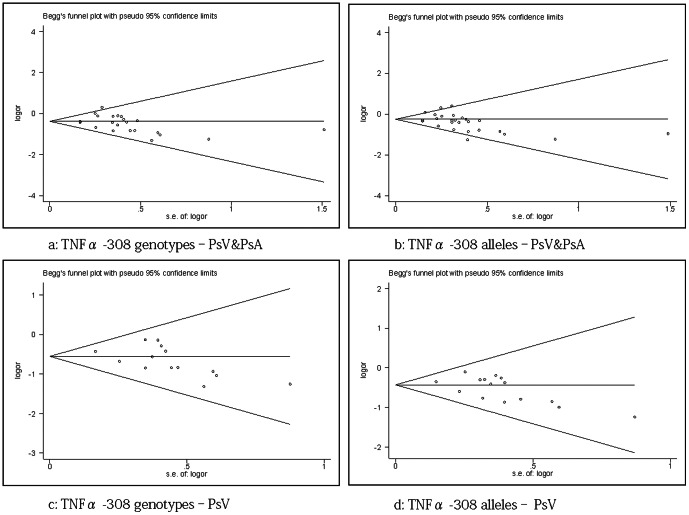
Funnel plot showing publication bias in 4 pooled groups of PsV & PsA and PsV only. The value of Pr>|z| (continuity corrected) is p<0.10.

For the genotypes and alleles of TNFα -308A/G, the publication bias showed statistical significance in the pooled groups of PsV & PsA (Begg’s test, p = 0.040, 0.106; Egger’s test, p = 0.021, 0.034 [separately]). Similarly, the same publication bias was found in the pooled groups of PsV (Begg’s test, p = 0.025, 0.054; Egger’s test, p = 0.070, 0.050 [separately]). The funnel plots for publication bias are shown in Figure 6 and suggest the evidence of potential publication bias. In all other groups, neither the Begg’s (p>0.1) nor Egger’s tests (p>0.1) showed statistical significance, which suggested that no publication bias existed (data not shown).

## Discussion

It is well known that TNFα plays a crucial role in autoimmune and infectious diseases as a proinflammatory cytokine; however, the mechanism of altered PsV & PsA risk owing to SNPs in the TNFα gene promoter region remains unknown. A possible explanation is the abnormal expression of the TNFα gene mediated by variations in the TNFα promoter. This premise was supported by studies noting that the TNFα -857C allele was associated with positive response to drug treatment in PsV patients treated with etanercept (Recombinant Human Tumor Necrosis Factor-α Receptorα:IgG Fc Fusion Protein)[43]. However, the connection between TNFα transcriptional activity or protein production and TNFα promoter SNPs is unresolved, as the reports are inconsistent with some studies revealing no association between them [44]. Therefore, more complex mechanisms than the abnormal expression of the TNFα gene must exist. Another possible explanation is that TNFα promoter SNPs is also associated with the production of other cytokines that play an important role in the pathologic process of the disease. For example, TNFα -308A allele was associated with higher serum interferon-alpha levels in the patients with juvenile dermatomyositis [45]. Furthermore, because of the complexity of biological systems, a single gene polymorphism may result in further polymorphisms or post-transcriptional regulation under different gene-gene or gene-environment interactions. Accordingly, further studies are needed to assess the functional consequences of TNFα promoter SNPs. In the present study, the variant genotypes and alleles of TNFα promoter SNPs were found to contribute to the same risk of disease susceptibility in all groups or subgroups.

It remains controversial whether PsV and PsA are different clinical manifestations of one disease or two different clinical entities. On the one hand, PsV and PsA exist unseparated in patients because of the complex and heterogeneous nature of PsV & PsA [16]. On the other hand, the diversity of skin lesions, including the presence and severity of a clinical rash, does not affect the clinical manifestation of joint disease, which is found in 30% of patients with skin lesions [4]. However, the common pathogenesis, activation of T lymphocytes and proinflammatory cytokines, is now well recognized in patients with PsV and/or PsA [16]. In addition, the curative effect of TNFα blockers in patients with PsV and/or PsA indicates that, to a certain degree, they share a common immune mechanism [5]. Therefore, both PsV and PsA may have a common onset risk with regard to TNFα promoter SNPs. Our meta-analysis results confirmed this hypothesis through the quantitative synthesis of controversial studies. The TNFα -857T/C variant significantly increased the susceptibility to PsV & PsA (OR = 1.536, 95% CI, 1.336-1.767), without heterogeneity among the groups. For TNFα -238A/G, a greater risk (OR = 2.493, 95% CI, 1.777-3.498) was found using the random-effects model, with heterogeneity primarily regarding the year of publication. However, the TNFα -308A/G variant exhibited a protective effect, with heterogeneity primarily in the disease subtype (OR = 0.682, 95% CI, 0.596-0.779). Further subgroup-analysis found the same risk or protective effect in most of the subgroups without heterogeneity. In a word, a variation in the levels of TNFα production attribute to a functional consequence of these TNFα promoter SNPs, which results to a common onset risk in both PsV and PsA.

Although a meta-analysis completed by Rahman et al. revealed that PsA risk was associated with TNFα -238A/G polymorphism (OR = 2.29; 95% CI 1.48-3.55) and not with TNFα -308A/G polymorphism (OR = 0.92, 95% CI, 0.69 -1.23) [13], more recent studies also paid close attention to other SNPs in the TNFα promoter, such as TNFα -857T/C [17]. Additionally, an increasing number of studies have provided new evidence for PsA susceptibility, although the results are controversial. Our meta-analysis gathered the comprehensive evidence and is the first to reveal that TNFα -857T/C polymorphism is associated with PsA susceptibility using the fixed-effects model (OR = 1.419, 95% CI, 1.214-1.658). Lv, K. et al. have observed in vitro that the TNFα -857T/C variant is a stronger transcriptional activator in response to lipopolysaccharide stimulation in the RAW264.7 cell line [46], indicating that the main mechanism of increased PsA risk can be attributed to the TNFα -857T/C polymorphism. Moreover, the results reflecting the association between TNFα -238A/G or TNFα -308A/G polymorphism and PsA susceptibility are remarkably consistent compared with the previous meta-analysis, although the pooled ORs differ because more studies and the fixed-effects model were used in our meta-analysis.

Furthermore, similar to a previous meta-analysis performed by Chunying Li et al. [11], our meta-analysis results showed that the TNFα -238A/G and TNFα -308A/G polymorphisms separately had the same risks and protective effects with regard to PsV susceptibility. However, in the subgroup-analysis, the results differ because recent studies have provided new evidence for the role of TNFα promoter SNPs in PsV susceptibility [15]. In the Asian subgroup, the TNFα -238A/G variant increased PsV risk by 1.912 (95% CI 1.047-3.491) using the fixed-effects model compared with the value of 1.46 (95% CI 0.66-3.19) revealed previously. However, no association was found between PsV risk and TNFα -238A/G or TNFα -308A/G polymorphism (OR = 1.569, 0.856, 95% CI 1.11-8.71, 0.523-1.400) using the fixed-effects model for the type II PsV subgroup compared to the previous Chunying Li et al. meta-analysis (OR = 3.11, 0.57, 95% CI, 0.800-3.078, 0.37-0.88). The inconsistency in the meta-analysis results indicates that the alterative risk of PsV result from TNFα promoter SNPs may be more complex because of the presence of stratification factors, such as ethnic admixture and the subtype or severity of disease.

Our meta-analysis has several limitations. First, heterogeneity was observed in some groups; although a subgroup analysis was performed, heterogeneity also existed with unclear sources in some subgroups. This finding indicated that the genetic background, combined with linkage disequilibrium patterns, epigenetic modifications, and clinical heterogeneity, made the relationship between the risk of disease susceptibility and TNFα promoter SNPs more complicated. Second, on analyses the risk of psoriasis with TNFα promoter SNPs, patients only with PsV and/or PsA were involved, but not with erythroderma or pustular lesions. Besides, only Caucasian and Asian patients were involved in the subgroup analysis; therefore, the assessment of disease risk is not applicable to other disease subtypes and ethnicities. Third, apart from the limited sample size or quality control of genotyping in some of studies, some subgroup analyses only involved three studies; this small sample size indicated insensitivity with regard to exploring the real association. Finally, as some negative-association studies are often unpublished, it seems unavoidable that publication bias may influence the meta-analysis results. Accordingly, caution should be exercised when interpreting the results of this meta-analysis.

## Conclusions

This meta-analysis confirms the previous results that TNFα -238A/G and TNFα -308A/G polymorphisms are associated with PsV or PsA susceptibility. Furthermore, this meta-analysis shows that the TNFα -857T/C variant increases the risk of PsA significantly, whereas TNFα -857T/C and TNFα -238A/G polymorphisms are risk effects in the pooled analysis of PsV & PsA. In contrast, the TNFα -308A/G polymorphism is a protective effect for PsV & PsA. No significant association between TNFα-308A/G, -1031C/T, or -863A/C polymorphism and PsA susceptibility was revealed by this meta-analysis. Thus, further studies are needed to elucidate the precise contribution of those SNPs to the pathogenesis of PsV& PsA by using proteome-wide analysis of SNPs (PWAS) [47]. Additionally, prospective studies on the response to the TNFα blockers in patients with PsV& PsA to their TNFα promoter SNPs are benefit for properly assessing the impact and value of TNFα blockers in patients.

## Supporting Information

Checklist S1 **PRISMA 2009 Checklist.**
(DOC)
